# Recovery of W(VI) from Wolframite Ore Using New Synthetic Schiff Base Derivative

**DOI:** 10.3390/ijms24087423

**Published:** 2023-04-18

**Authors:** Rawan E. Elbshary, Ayman A. Gouda, Ragaa El Sheikh, Mohammed S. Alqahtani, Mohamed Y. Hanfi, Bahig M. Atia, Ahmed K. Sakr, Mohamed A. Gado

**Affiliations:** 1 Department of Chemistry, Faculty of Pharmacy, Heliopolis University, El Salam City, Cairo 11785, Egypt; 2 Department of Chemistry, Faculty of Science, Zagazig University, Zagazig 44519, Egypt; 3 Radiological Sciences Department, College of Applied Medical Sciences, King Khalid University, Abha 61421, Saudi Arabia; 4 BioImaging Unit, Space Research Centre, University of Leicester, Michael Atiyah Building, Leicester LE1 7RH, UK; 5 Research Center for Advanced Materials Sciences (RCAMS), King Khalid University, Abha 61413, Saudi Arabia; 6 Nuclear Materials Authority, El Maadi, Cairo P.O. Box 530, Egypt; mokhamed.khanfi@urfu.ru (M.Y.H.);; 7 Institute of Physics and Technology, Ural Federal University, St. Mira, 19, 620002 Yekaterinburg, Russia; 8 Department of Civil and Environmental Engineering, Wayne State University, 5050 Anthony Wayne Drive, Detroit, MI 48202, USA

**Keywords:** adsorption, Schiff base, tungsten, synthesis, kinetics

## Abstract

A new synthetic material, namely, (3-(((4-((5-(((S)-hydroxyhydrophosphoryl)oxy)-2-nitrobenzylidene) amino) phenyl) imino) methyl)-4-nitrophenyl hydrogen (R)-phosphonate)), was subjected to a quaternary ammonium salt and named (HNAP/QA). Several characterizations, such as FTIR spectrometry, ^1^H-NMR analysis, ^13^C-NMR analysis, ^31^P-NMR Analysis, TGA analysis, and GC-MS analysis, were performed to ensure its felicitous preparation. HNAP/QA is capable of the selective adsorption of W(VI) ions from its solutions and from its rock leachate. The optimum factors controlling the adsorption of W(VI) ions on the new adsorbent were studied in detail. Furthermore, kinetics and thermodynamics were studied. The adsorption reaction fits the Langmuir model. The sorption process of the W(VI) ions is spontaneous due to the negative value of ∆*G*° calculated for all temperatures, while the positive value of ∆*H*° proves that the adsorption of the W(VI) ions adsorption on HNAP/QA is endothermic. The positive value of ∆*S*° suggests that the adsorption occurs randomly. Ultimately, the recovery of W(IV) from wolframite ore was conducted successfully.

## 1. Introduction

The potential toxicity of heavy metals in aquatic environments varies significantly between their different chemical forms and the fact that they are neither biodegradable nor photodegradable [1]. As a lithophile element, tungsten can be found in wolframite and scheelite ores, where it is recovered as tungstate (WO_4_^2−^) and naturally occurs in soils and sediments [2,3]. The concentration of W(VI) in the lithosphere varies from 0.2 to 2.4 mg kg^−1^ [2,3,4], while in ocean water it is present in the range of 8.0 to 100 mg L^−1^. On the other hand, in the surface rocks of the Northern Atlantic and the Pacific Oceans, it is in the range of 1.0 to 1.3 mg kg^−1^ [2,4]. In terms of solubility and dominance, tungstate (WO_4_^2−^) predominates over its hydroxyl counterpart. The oxidation state of tungsten ranges from −2 to +6, making it a transition metal. There is a potential for tungstate species to polymerize in mildly acidic conditions, yielding iso-polytungstates, polytungstates, and monotungstates [5]. For example, tungsten inhibits molybdenum metabolism, which, in turn, reduces the biological activity of molybdenum-containing enzymes such phosphatase and adenosine triphosphate enzymes [6]. Even in tiny quantities, tungstate species present a significant hazard. As a result, testing and treating natural water supplies for tungsten is essential [7,8,9]. The disposal of their waste requires proper measurements, as this waste also contains valuable metals, and tossing these heavy metals is perilous due to the depletion of their primary resources, which leads to environmental hazards because of these minerals. Manganese is an important metal that has numerous applications in industry. It should be reprocessed and not disposed of. The high melting point of tungsten has extensive applications, such as manufacturing super alloys for high-temperature tasks, catalysts for redox chemical reactions, and fertilizers and in power plants as a regulator of oxygen and nitrogen compounds to mitigate environmental damage [10,11,12,13]. A hydrometallurgical process is used for the extraction of tungsten from both its ores and industrial effluents. Tungsten is recovered using processes such as solvent extraction [14,15], ion-exchange method [16], chemical precipitation [17], filtration [18], electrolysis and electro-deposition [19].

Tungsten is one of the elements classified as a significant raw material by the European Commission, according to its economic significance and supply chain. In 2022, the average price of tungsten was around USD 270 per metric ton unit of tungsten trioxide [20]. Tungsten has many extensive applications in several industries, such as the automotive industry, defense equipment, chemical, and aviation industries [21]. However, there are stringent restrictions for the production of tungsten in which Mo, V, Si, and P are frequently found in the products as contaminants which violate the European standards of tungsten purity. When manufacturing high-purity tungsten and tungsten products, molybdenum is one of the most difficult contaminants to remove because of its chemical similarity to tungsten [22]. It is common for molybdenum minerals to be found around wolframite and scheelite minerals. According to a previous study from China, the mass ratio of Mo/WO_3_ was about 2.0% in W-Mo-Bi-Sn scheelite crystals in the Shizhuyuan mine, while the mass ratio was above 5.0% in Mo‒W scheelite middlings from the Sandaogou mine [23].

The novelty of this work is the synthesis of a new adsorbent material with a high selectivity of W(VI) ions from its aqueous solutions and rock leachate. The prepared material undergoes several characterizations to ensure its successful preparation. The novel adsorbent is used for sorption of W(VI) ions from a synthetic solution and is finally used for the recovery of W(VI) ions from wolframite ore leachate via sorption and precipitation in a pure product.

## 2. Result and Discussions

### 2.1. Characterizations of the Sorbent

#### 2.1.1. FTIR Analysis

**Figure 1:**
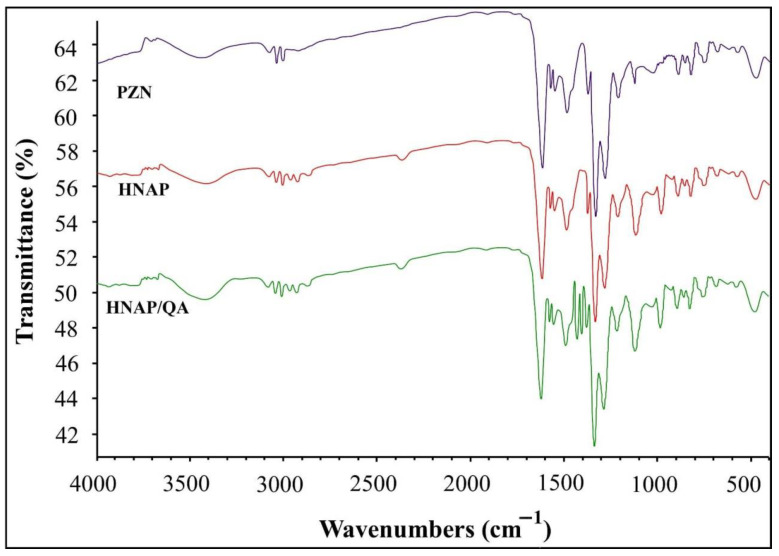
FTIR spectra of PZN, HNAP, and HNAP/QA.

The FTIR spectra of the prepared materials were collected and are presented in Figure 1. The broad peaks at 3385 and 3156 cm^−1^ are due to the vibrations of –NH and –OH groups, respectively. The band observed at 1588 cm^−1^ is related to the imine bond C=N, which is the most significant identification of the Schiff base [24]. The intense assignments located at 3049–3063 and 2987–3005 cm^−1^ are attributed to aromatic and aliphatic υ(C–H), respectively [25,26]. The N–O stretching vibrations in the nitro group occur nearly at 1538 and 1328 cm^−1^ of the asymmetrical and symmetrical vibrations, respectively. After phosphorylation, the composition of the HNAP was validated by FTIR, and new bands appearing at 2349 cm^−1^ are assigned to the P–H stretching mode. The assignment at 1170 cm^−1^ corresponds to the P=O stretching vibration. Moreover, a P–OH stretching vibration is recorded at 990 cm^−1^ [27]. The quaternization reaction (a reaction with glycidyl trimethyl ammonium chloride) brings numerous –CH_3_ groups with which a symmetric stretching vibration appears at 2875 cm^−1^ [28]. In addition, the two sharp features located at 1463 and 1409 cm^−1^ are assigned to the C–H stretching mode of the quaternary ammonium salt. The peak obtained at 964 cm^−1^ is due the asymmetrical stretching vibration of the quaternary nitrogen of quaternary ammonium salt [29].

#### 2.1.2. BET Surface Analysis

**Figure 2:**
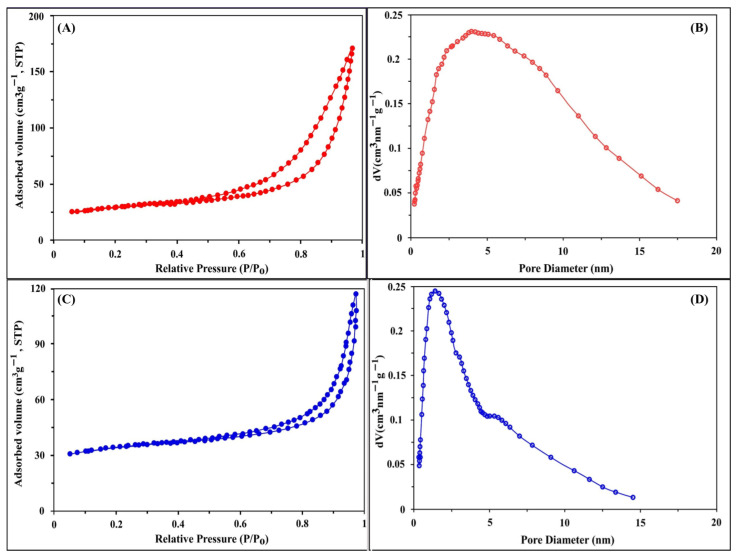
(**A**,**C**) N_2_ sorption/desorption isotherms of HNAP and HNAP/QA, and (**B**,**D**) pore volume distribution of HNAP and HNAP/QA.

The surface area (S_BET_) and porosity of HNAP and HNAP/QA were detected using the Brunner–Emmett–Teller theory at 77K. As seen in Figure 2, the N_2_ sorption/desorption curves of HNAP and HNAP/QA are defined as IV type isotherms with limited H_3_-type hysteresis. This means that HNAP and HNAP/QA are mesoporous-type materials. The HANP/QA surface area (S_BET_: 178.4 m^2^ g^−1^) is higher than that of the HANP surface area of 101.6 m^2^ g^−1^. In addition, there is a slight increase in the pore volume of HNAP, increasing partly from 0.205 cm^3^ g^−1^ for HNAP to 0.288 cm^3^ g^−1^ for HNAP/QA after a reaction with glycidyl trimethyl ammonium chloride (Table 1).

#### 2.1.3. ^1^H-NMR Analysis

**Figure 3:**
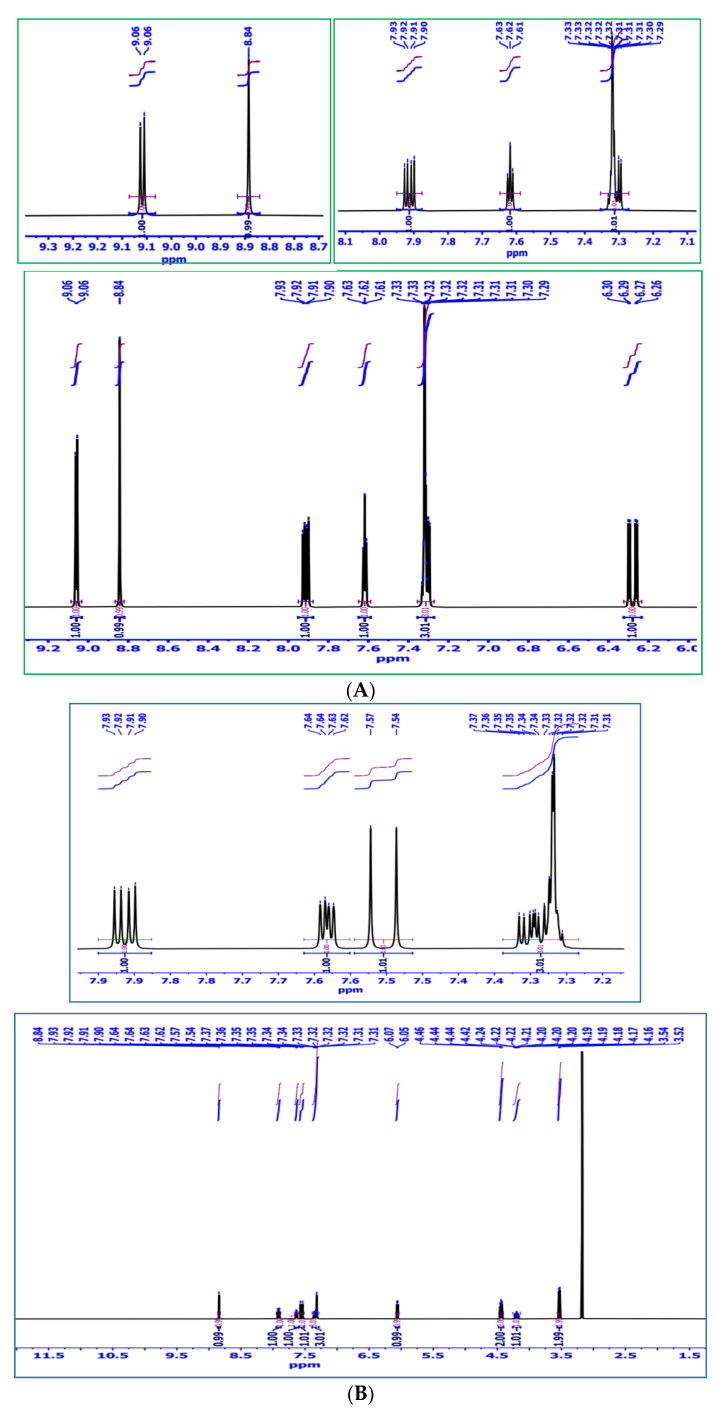
^1^H-NMR spectra of (**A**) HNAP and (**B**) HNAP/QA.

^1^H-NMR analysis is considered an efficient technique that provides significant information about the protons of the synthetic adsorbent to determine its structure. The principal assignments (δ) observed at 7.31–7.91 ppm are assigned to the protons of –CH in the benzene ring. The assignments at 9.06, 8.84, and 6.28 ppm are due to the protons of –OH, –NH, and –PH, respectively. The characterization of HNAP is illustrated in Figure 3. After modification, significant differences are observed in HNAP/QA spectrum. The chemical shift of the –OH group appears as a doublet at 6.06 ppm with a coupling constant of J = 5.74 Hz. A shift to a lower chemical shift (7.55 ppm, d, 1H, J = 14.3 Hz) is observed for –the PH group due to the modification. Furthermore, new assignments were observed for methyl groups (3.18 ppm, s, 3H, J = 8.2 Hz), methine groups (4.2 ppm, m, 1H, J = 7.46 Hz), and aliphatic methylene groups (4.44 ppm, m, 2H, J = 11.38 Hz).

#### 2.1.4. ^13^C-NMR Analysis

**Figure 4:**
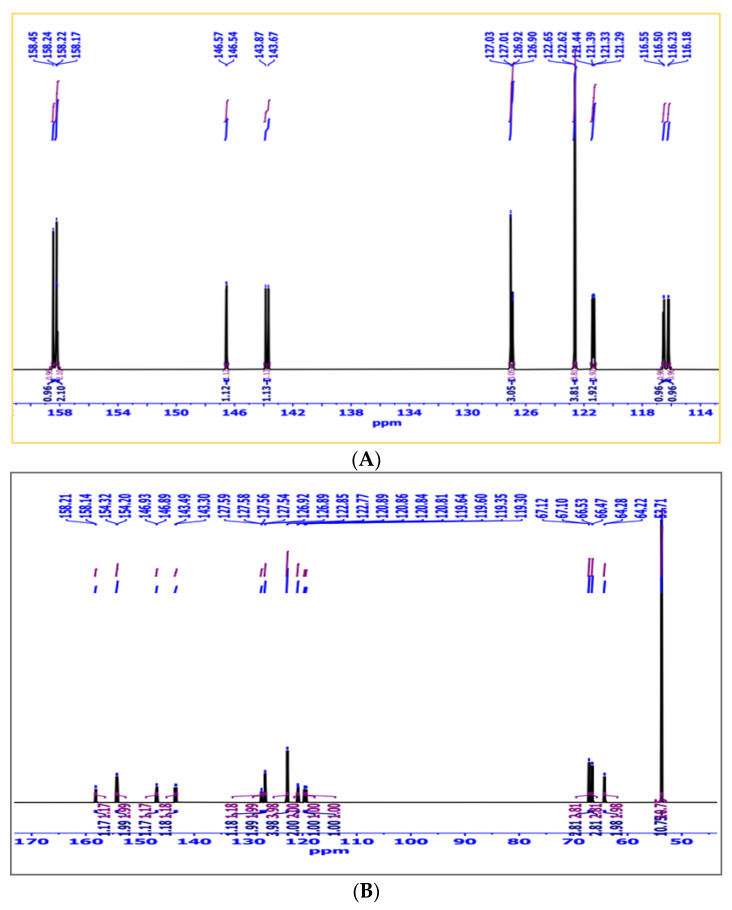
^13^C-NMR analysis of (**A**) HNAP and (**B**) HNAP/QA.

A ^13^C-NMR analysis of both HNAP and HNAP/QA was applied at 100.01 MHz to investigate the number of carbon atoms. As demonstrated in Figure 4A, the main assignments δ located around 126.9–127.5 ppm (s, J = 11.5, 6.9 Hz) are related to the carbon atom attached to the carbon of the imine group, the features at 146.6 ppm (s, J = 11.7, 7.9 Hz, –C=N) are related to the carbon of the imine group, the assignments at 143.8 ppm (s, J = 19.5 Hz) are attributed to the carbon attached to the nitro groups, and the assignments at 158.2 ppm (s, J = 7.5 Hz) are due to the carbon of the benzene ring attached to the phosphonic acid group, while the carbons of the benzene ring show assignments at 116.2–127 ppm (s, J = 3 Hz). Interestingly, the assignments of the de-protonated carbons were shown to be higher when de-shielded than any protonated carbons. After modification, a significant difference is observed in HNAP/QA, as illustrated in Figure 4B. A new feature appearing at 66.5 ppm (s, J = 6, 3.7 Hz, –CH_2_) is related to the methylene group attached to the oxygen of the phosphonic acid group, the assignment at 64.2 ppm (s, J = 6.1 Hz, –CH) is related to the carbon attached to the –OH group, the assignment at 67.1 ppm (s, J = 6.1 Hz, –CH_2_) is due to the carbon attached to the nitrogen of the quaternary nitrogen group, and finally, the carbons of the methyl groups of the quaternary nitrogen group show assignment at 53.7 ppm (s, J = 4.6 Hz, –CH_3_). 

#### 2.1.5. ^31^P-NMR Analysis

**Figure 5:**
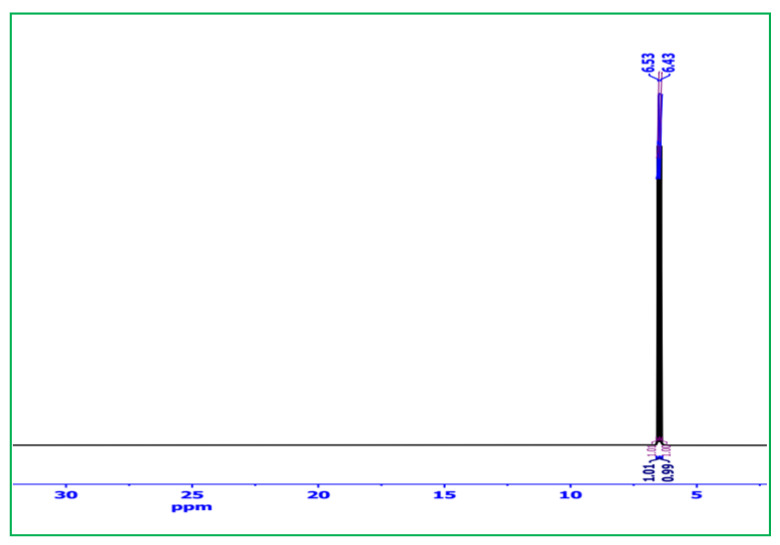
^31^P-NMR analysis of HNAP/QA.

A ^31^P-NMR analysis with an energy of 200.01 MHz is presented in Figure 5. The primary assignments of the HNAP/QA phosphorus atoms are observed at 6.53 ppm as singlets. 

#### 2.1.6. GC-MS Analysis

**Figure 6:**
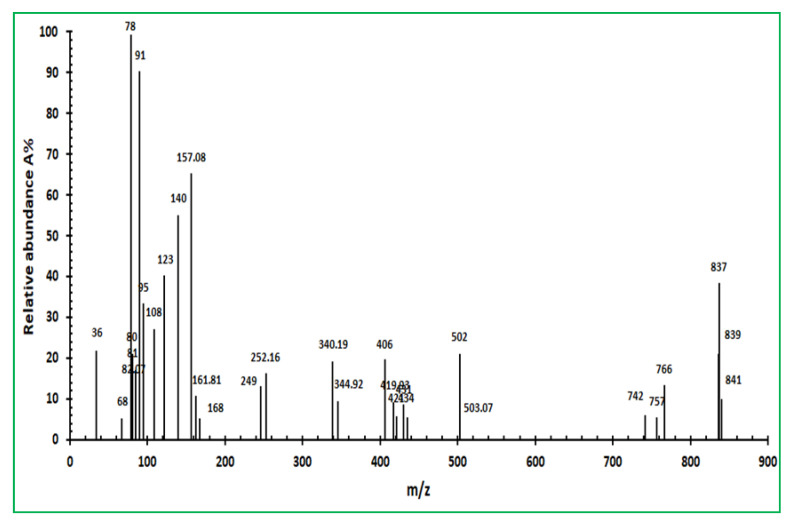
GC-Mass analysis of HNAP/QA.

Figure 6 depicts the GC-MS chromatogram of HNAP/QA. It reveals that the most stable fragment [m/z]^+^ is [C_32_H_44_N_6_P_2_O_12_Cl_2_]˙, with a molecular weight of 837.58 g/mol and a relative abundance of 35%. The obvious peak at 78 g/mol with a relative abundance of 99% is due to the [C_6_H_6_]˙ benzene ring [C_6_H_6_]˙ or the [C_3_H_7_Cl]˙ propyl chloride. The fragments of nitro benzene [C_6_H_5_NO_2_]˙, tropolium cation [C_7_H_7_]˙, and para-phenylene diamine [C_6_H_8_N_2_]˙ have molecular weights of 123, 91, and 108 g/mol. The tiny peak at 64 g/mol with a relative abundance of 5% illustrates the formation of an ethyl chloride fragment [C_2_H_6_Cl]˙. The molecular weight of 36 g/mol is related to the [HCl]˙ fragment, which explains the liberation of hydrogen chloride gas. Moreover, the molecular weight of 95 g/mol refers to the formation of the quaternary trimethyl ammonium chloride moiety [C_3_H_10_NCl]˙. The recorded fragment with a molecular weight of 140 g/mol is due to para nitro phenol [C_6_H_5_NO_2_]˙. An important note should be made for the fragmentation pattern mechanism for the compounds containing chlorine atoms. This fragmentation mechanism is most important where chlorine is a good leaving group. When one chlorine atom is present, M and M + 2 isotopic peaks become very significant, where M represents the molecular ion peak (M = 837 with an A% of 35% and M + 2 = 839 with an A% of 21). If the compound contains two chlorine atoms, a M + 4 isotopic peak should be observed, as well as an intense M + 2 peak (M + 4 = 841 with an A% of 10%). In addition, the thermal analysis of HNAP/QA is introduced in a supplementary file Figure S1.

### 2.2. Sorption of W(VI) Ions

Different factors were studied to optimize the sorption of W(VI) ions on the HNAP/QA ligand, as described below.

#### 2.2.1. Influence of pH on W(VI) Ion Sorption

**Figure 7:**
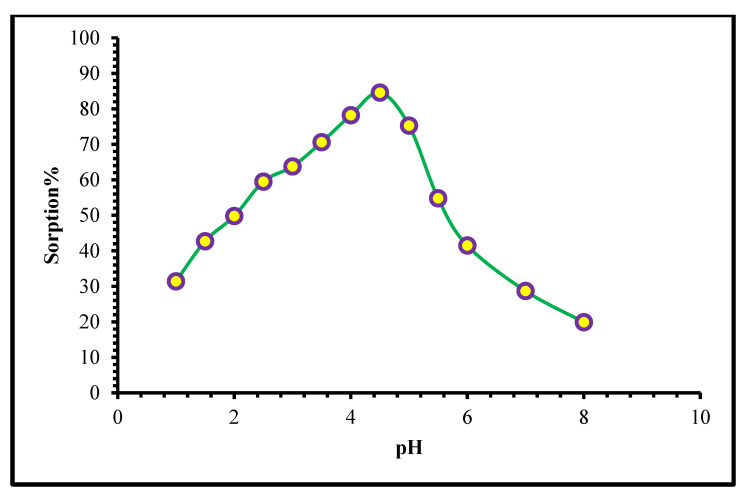
Effect of the solution pH on the adsorption % of W(VI) using HNAP/QA (W(VI) = 250 mg L^−1^, 20 mL solution, 30 min, room temperature).

It is noteworthy that the pH value of the aqueous solution has a crucial impact on the adsorption of W(VI) ions. Changing the pH value has an obvious influence on not only the protonated/deprotonated active sites of the HNAP/QA but also the speciation of the tungsten ions formed. The pH value and metal content can affect the formation of W(VI) ions, as reported previously in in the literature [30]. Tungsten is a predominantly anionic species at pH values of over 2.0. Tungsten’s cationic species are completely absent at these pH levels. Since most W(VI) is found in anionic species, the adsorption behavior of W(VI) ions with HNAP/QA was studied in relation to the pH value. Given that WO_3_(s) formation is possible at pH values below 2.0 in solutions [31], the obtained data (shown in Figure 7) demonstrate a general observed trend that the adsorption of W(VI) ions increased gradually from a pH 1.0 to 4.0 and reached a maximum adsorption efficiency at pH 4.5. The W(VI) ions sorption then decreased as the pH increased past a value of 4.5. At a pH value of 4.5, the percentage of absorbed tungsten reached a plateau of greater than 80% and then decreased gradually as a function of pH between pH 5.0 and pH 8.0. Understanding the tungsten solution species helps interpret this pH change.

For pH values between 2.0 and 8.0, W(VI) ions are present in the form of polynuclear anionic species, such as [W_12_O_39_]^−^, [W_12_O_41_]^10−^, and [W_6_O_20_(OH)]^5−^, while the formation of a neutral species, WO_2_, occurs at higher pH values. As the pH of a solution rose from 7.5 to 9.0, the adsorption percentage of W(VI) decreased. Furthermore, the active site of the HNAP/QA sorbent is the quaternary ammonium chloride moiety, which acts as anion exchanger. This can be explained by a shift in the species distribution. A pH of 4.5 is the most suitable pH for the adsorption of W(VI) ions in this study.

#### 2.2.2. Effect of HNAP/QA Dose

**Figure 8:**
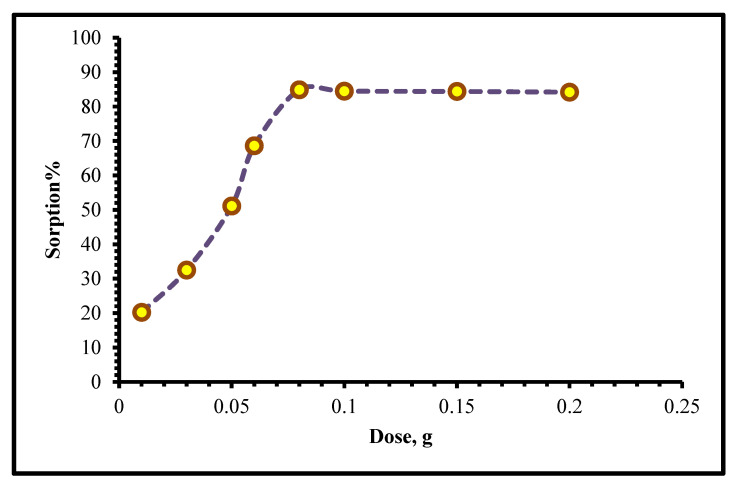
Effect of the HNAP/QA dose on the adsorption % of W(VI) (pH 4.5, W(VI) = 250 mg L^−1^, 20 mL solution, 30 min, room temperature).

Figure 8 shows the impact of HNAP/QA dosage on the sorption percentage of W(VI) ions. The experiment was applied by boosting the adsorbent quantity from 0.01 to 0.2 g while holding all other variables stable at a 250 mg L^−1^ W(VI) ion concentration and room temperature. High concentrations of HNAP/QA expose more of the tungsten’s surface, leading to a higher rate of adsorption. The adsorption was improved noticeably as the dose was raised from 0.01 to 0.08 g. There was a little noticeable change in the adsorption process after adding more adsorbents. The percentage of metal adsorbed determines the sorption capacity of HNAP/QA, and the data show that the greatest adsorption efficiency of tungsten was achieved at 0.08 g of HNAP/QA.

#### 2.2.3. Kinetics Study

**Figure 9:**
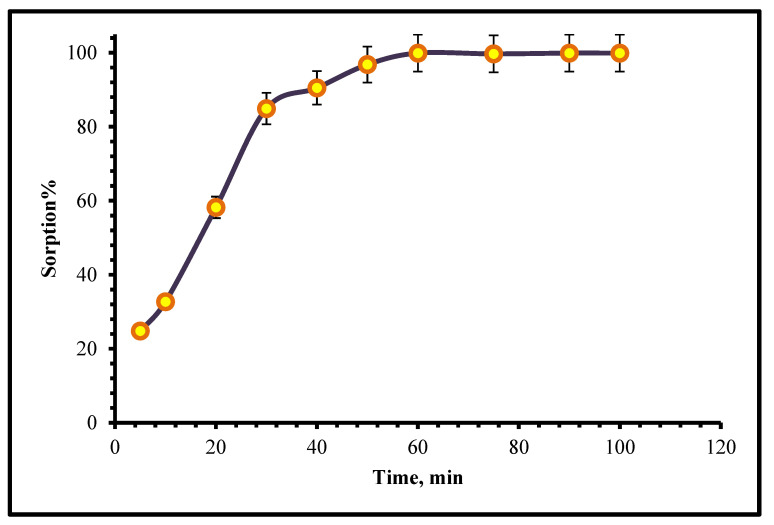
Effect of the agitation time on the adsorption % of W(VI) using HNAP/QA (pH 4.5, W(VI) = 250 mg L^−1^, 20 mL solution, 0.08 g, room temperature).

By analyzing kinetic profile, we can learn not only how long it takes to reach equilibrium but also how stable the material is, how it interacts with the target solute, and which stage in the sorption mechanism is ultimately responsible for regulating the process [32]. In fact, sorption occurs on the easily accessible active sites at the surface of the sorbent and the first exterior layers of the material, and almost 84% of total sorption occurs within the first 30 min of contact. The second stage involves a more gradual sorption caused by diffusion inside the sorbent’s pores. As presented in Figure 9, after 60 min of agitation, the equilibrium time was achieved because the WO_4_^2−^ ions were adsorbed continuously from the solution until equilibrium was accomplished, and then no further change was observed after the increase in time. 

**Figure 10:**
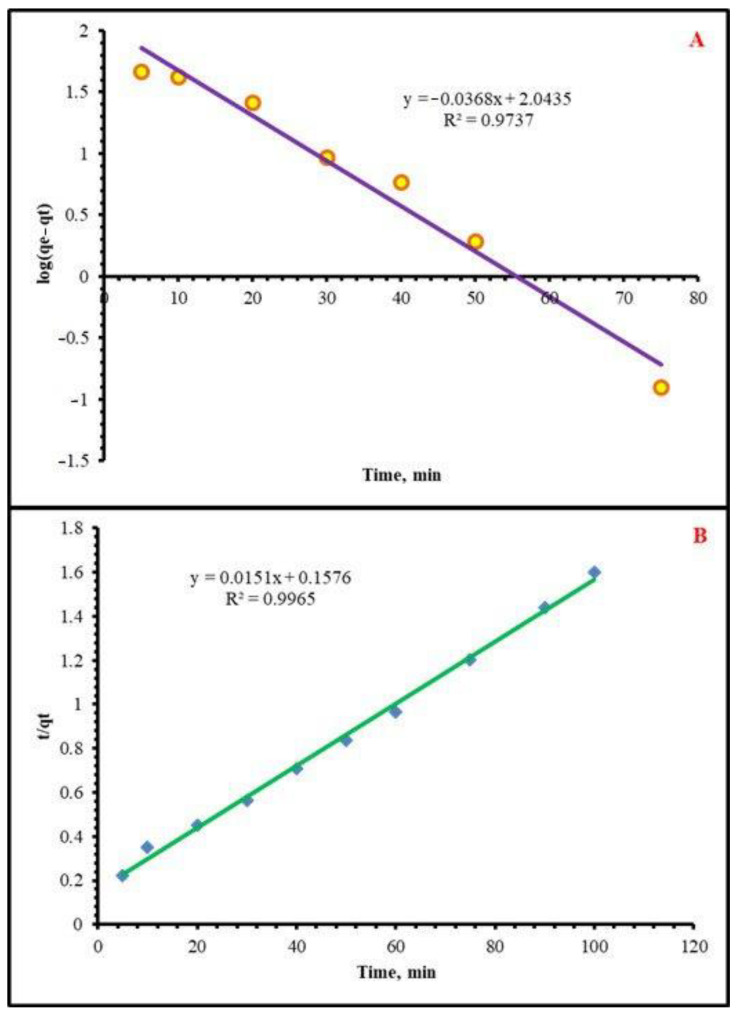
(**A**) PFO and (**B**) PSO kinetics of W(VI) ions absorbed on HNAP/QA.

The linear forms of Lagergren’s pseudo-first-order (PFO) and second-order (PSO) rate expression were used to predict the order of the kinetic adsorption process of W(VI) ions on HNAP/QA, as presented in equations below [33,34,35,36]:

(1) $$\log(q_{e}-q_{t})=\log q_{e}-\frac{K_{1}t}{2.303}$$

(2) $$\frac{t}{q_{t}}=\frac{t}{q_{e}}+\frac{1}{K_{2}q_{e}^{2}}$$

where *K*_1_ represents the PFO rate constant (min**^−^**^1^), *K*_2_ is defined as the rate constant of the PSO (g mg**^−^**^1^ min**^−^**^1^), and the quantities of WO_4_^2−^ ions sorbed at any moment are denoted by *q_t_* (mg g**^−^**^1^). As provided in Figure 10, the log(*q_e_* − *q_t_*) vs. the agitation time (*t*) shows that the PFO model does not match the adsorption of WO_4_^2−^ species on HNAP/QA. The calculated *q_e_* value is 110.535 mg g**^−^**^1^ (Table 2), higher than the value of the experimental *q_e_* (62.44 mg g**^−^**^1^). Nonetheless, the PSO model has a significantly higher value of R^2^ (0.9965), which is close to unity, as listed in Table 2. Furthermore, the calculated *q_e_* value, 66.225 mg g**^−^**^1^, is also very close to the experimental value of *q_e_*, 62.44 mg g**^−^**^1^. The data point to a better match of the PSO equation to the adsorption of WO_4_^2−^ ions on HNAP/QA. Thus, the chemical adsorption process of sharing/exchanging electrons between WO_4_^2−^ species and HNAP/QA is best described as pseudo-second-order kinetics [37,38,39]. 

#### 2.2.4. Effect of the Initial W(VI) Concentration

**Figure 11:**
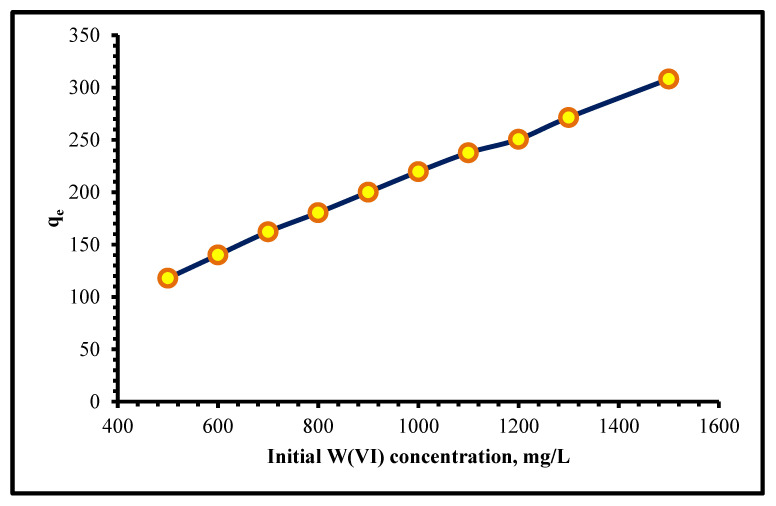
Effect of initial W(VI) ions concentration on the adsorption % of W(VI) using HNAP/QA (pH 4.5, 20 mL solution, 30 min, 0.08 g, room temperature).

With the aim of determining the distribution of W(VI) ions between the aqueous and HNAP/QA phases, a study was conducted at 25 °C with 20 mL of several solutions of W(VI), ranging in initial concentrations from 250 to 1500 mg L^−1^, and 0.08 g of HNAP/QA for 60 min. Figure 11 illustrates that the *q_e_* of HNAP/QA rises regularly as the concentration of W(VI) ions increases. As a result, the optimal starting HNAP/QA adsorbent concentration in this study was determined to be 1250 mg/L.

#### 2.2.5. Isotherm Study

**Figure 12:**
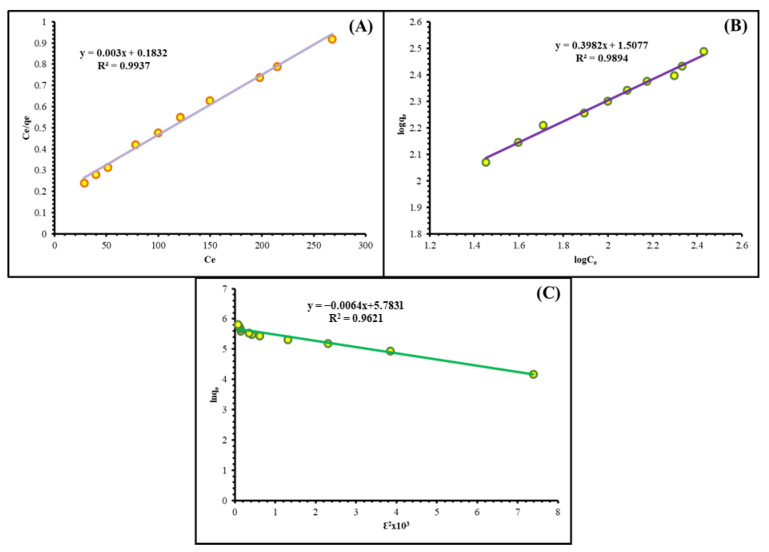
(**A**) Freundlich, (**B**) Freundlich, and (**C**) Dubinin–Radushkevich isotherm models of W(VI) ions adsorbed on HNAP/QA.

Isotherm models representing the equilibrium distribution between the liquid and solid phases of the sorption system were attained. Langmuir, Freundlich, and Dubinin–Radushkevich isotherm models provide a valuable insight into the affinity of the HNAP/QA sorbent to the target W(VI) ions, the maximal sorption capacity at which the sorbent is saturated, and the binding mechanism between the W(VI) ions and HNAP/QA. Initially, the Langmuir isotherm model is expressed using the given equation below [40,41]:

(3) $$\frac{C_{e}}{q_{e}}=\frac{C_{e}}{q_{m}}+\frac{1}{q_{m}K_{l}}$$

where *C_e_* symbolizes the concentration at equilibrium (mg L^−1^), *q_e_* and *q_m_* represent the amount adsorbed and the maximal amount adsorbed (mg g^−1^), and *K*_1_ refers to the apparent heat change. The Langmuir equation assumes a uniform sorption energy, and sorption occurs as a monolayer devoid of sorbent molecule interactions. As provided in Figure 12A and Table 3, the adsorption of W(VI) ions on HNAP/QA is shown to be Langmuir-like, indicating a monolayer of W(VI) ions covering the surface of HNAP/QA; the R^2^ value approaches 0.99, and the Langmuir equilibrium constant *K_l_* has a value of 0.0164 L mmol^−1^. The Langmuir model predicts a maximum adsorption capacity of W(VI) ions on HNAP/QA of 333.33 mg g^−1^, which is very close to the experimental adsorption capacity of 326.75 mg g^−1^.

Secondly, the Freundlich isotherm denotes the multilayer sorption of ions onto the surface of the sorbent material, as described in the equation below [42,43]:

(4) $$\log q_{e}=\log K_{f}+\frac{1}{n}\log C_{e}$$

where *K_f_* corresponds to the adsorption capacity of HNAP/QA (mg g^−1^) and *n* is a constant. The smaller the 1/*n* value, the stronger the interaction between the metal ions, the adsorbent and the working adsorbate. Additionally, 1/*n* = 1 signifies linear adsorption, which results in the same adsorption energies across all sites. According to the Freundlich isotherm, the binding strength decreases when the number of binding sites is increased [44]. The data in Table 3 and the plot in Figure 12B reveal that the Freundlich isotherm does not agree with the sorption of W(VI) ions on HNAP/QA, for which the *K_f_* value (32.189 mg g^−1^) is significantly lower when compared with the *q_e_*_(exp)_.

Lastly, the Dubinin–Radushkevich isotherm model is applied to differentiate between physical and chemical sorption because of adsorption heterogeneity [45,46,47]. It calculates the surface energy heterogeneity. The following is a linear formula of the Dubinin–Radushkevich model, calculated from the following equations:

(5) $$\ln q_{e}=\ln q_{D}-B_{D}{\left(\varepsilon \right)}^{2}$$

(6) $$\varepsilon =RT\ln(1+\frac{1}{C})$$

where *B_D_* (mol^2^ kJ^−2^) represents the energy of sorption, and ε symbolizes the Polanyi potential. *B_D_* offers an opportunity to determine the adsorption energy *E* (kJ mol^−1^) of W(VI) ions on HNAP/QA [48].

(7) $$E=\frac{1}{\sqrt{2B_{D}}}$$

If the calculated value of *E* is less than 8.0 kJ mol^−1^, the sorption process is defined as physisorption, while if the value of *E* is 8.0 < *E* < 16.0 kJ mol^−1^, the sorption is believed to be chemisorption [49,50]. The data obtained from Figure 12C and listed in Table 3 show the *E* value is 8.839 kJ mol^−1^. Therefore, the sorption of W(VI) ions on HNAP/QA is chemisorption process. The result is convenient with the Langmuir isotherm, which suggests a monolayer sorption.

#### 2.2.6. Thermodynamics Study

**Figure 13:**
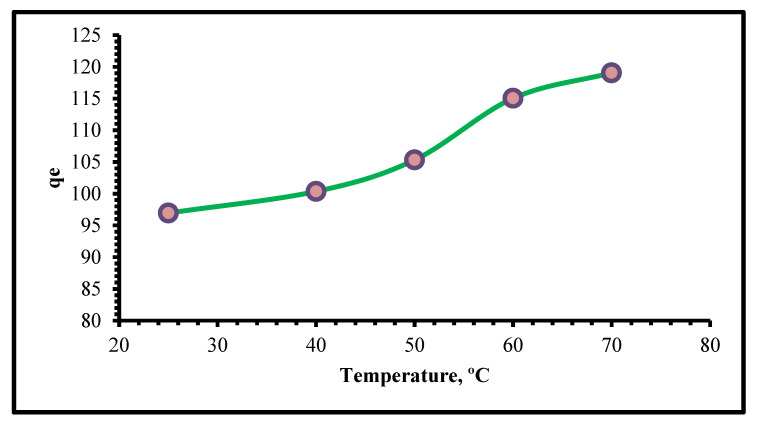
Effect of temperature on the adsorption % of W(VI) using HNAP/QA (pH 4.5, W(VI) = 250 mg L^−1^, 20 mL solution, 0.08 g).

The impact of temperature on the adsorption process was investigated using a 1250 mg L^−1^ W(VI) solution, a pH value of 4.5, and 0.08 g (dry mass) of HNAP/QA at 298, 313, 323, 333, and 343 K. As demonstrated in Figure 13, the reaction rate rose gradually when the temperature was increased. As a result, the adsorption is considered an endothermic process; therefore, increasing the ambient temperature of the system is the correct choice to speed up the adsorption.

The thermodynamic parameters controlling the sorption system (Δ*G*°, Δ*H*°, and Δ*S*°) were mathematically determined from the Van ’t Hoff equations as follows [51,52]:

(8) $$logKd=\frac{\Delta S}{2.303R}-\frac{\Delta H}{2.303RT}$$

**Figure 14:**
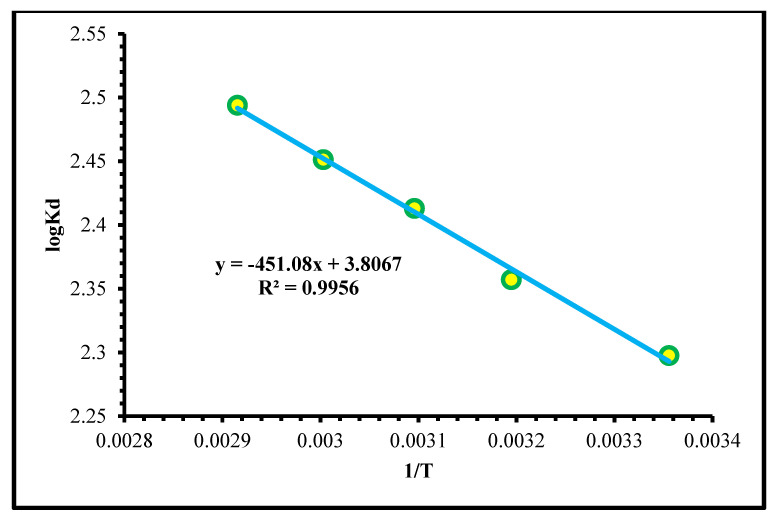
Plot of log *K_d_* vs. 1/*T* of W(VI) ions adsorbed on HNAP/QA.

(9) ΔG°=ΔH°−TΔS°

where *T* is a symbol of the temperature of the system (*K*), and *R* stands for the gas constant (8.314 J mol^−1^ K^−1^). Both ∆*H*° and ∆*S*° can be calculated from the slope and the intercept of log *K_d_* against the 1/*T* plot, as illustrated in Figure 14. The correlation coefficient is valued at R^2^ = 0.9956. The values of the thermodynamic parameters are tabulated in Table 4; the negative value of ∆*G*° for all temperatures proves that the adsorption of W(VI) ions on HNAP/QA occurs spontaneously. In addition, the positive values of ∆*H*° and ∆*S*° indicate the endothermicity and randomness of the W(VI) sorption process.

#### 2.2.7. Effect of Foreign Metal Ions on Selectivity

**Figure 15:**
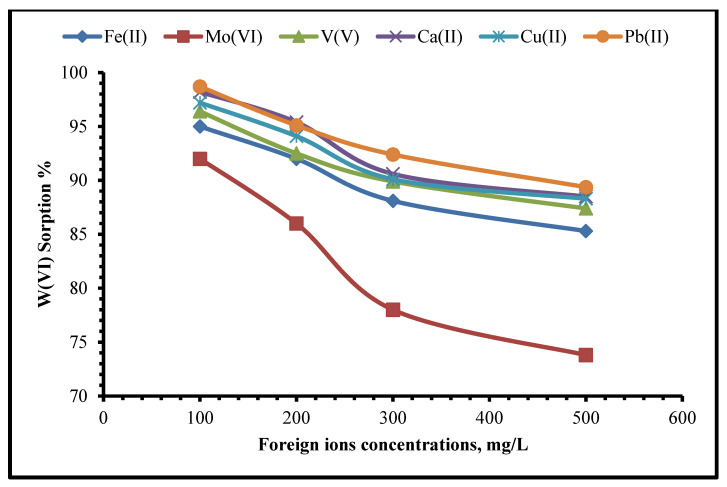
Effect of foreign ions on the adsorption % of W(VI) using HNAP/QA (pH 4.5, 20 mL solution, 30 min, 0.08 g, room temperature).

In order to investigate the selectivity of HNAP/QA toward the uptake capacity of W(VI) ions in the presence of different existing metal ions, the sorption process of W(VI) ions was conducted employing several ion solutions of Mo(VI), V(V), Ca(II), Cu(II), and Pb(II) with different concentrations ranging between 10 and 500 mg L^−1^. The obtained data in Figure 15 illustrate that the selectivity strength of the HNAP/QA material was slightly affected by the high concentrations of V(V), Ca(II), Cu(II), and Pb(II) ions. Impressively, the uptake of W(VI) ions was intensely influenced by the presence of Mo(VI) ions. It is more likely that the ability of the HNAP/QA material to adsorb cations through the chelation mechanism with a lone pair of electrons on the hydroxyl groups as well as the phosphine group of oxygen results in a reduction in the selectivity of the HNAP/QA sorbent to some extent. In the case of Mo(VI), both metals have the same chemical properties; therefore, both cations compete for the same binding sites, causing an obvious decrease in the adsorption efficiency. However, the HNAP/QA adsorbent is still effective in the case of the presence of several ions in the system. The result indicates that the HNAP/QA adsorbent demonstrates a high selectivity of W(VI) ions compared to other elements.

### 2.3. Desorption–Regeneration Study

**Figure 16:**
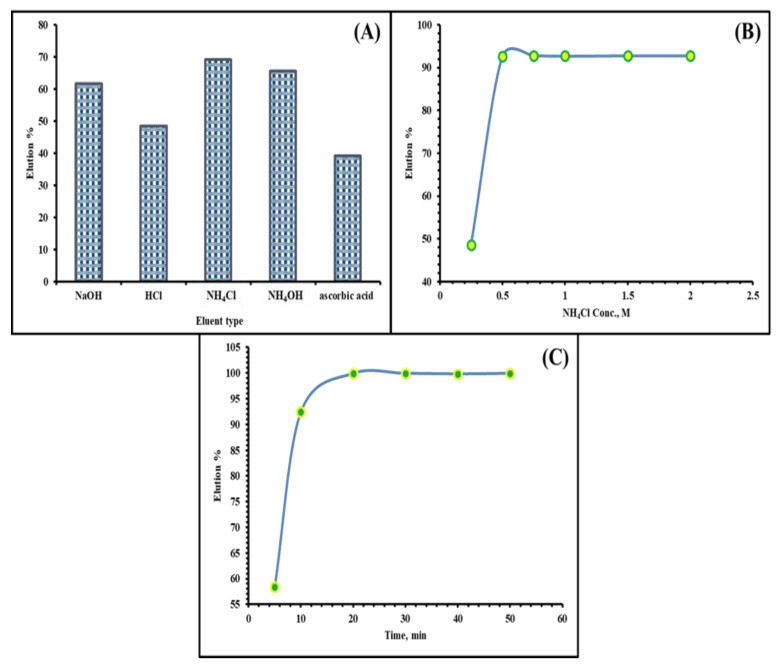
Factors affecting the desorption of W(VI) from HNAP/QA: (**A**) eluent type, (**B**) eluent concentration, and (**C**) elution time.

**Figure 17:**
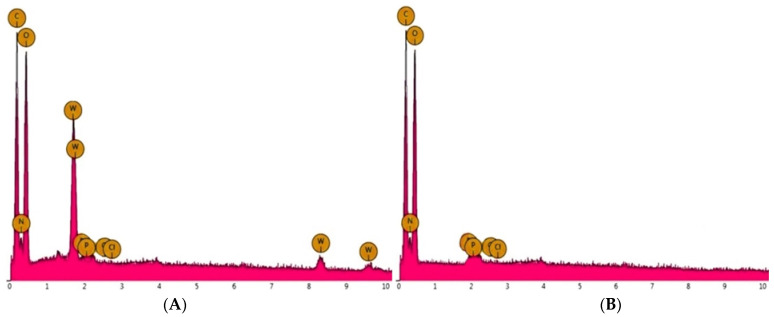
EDX analysis of (**A**) pregnant HNAP/QA with W(VI) and (**B**) HNAP/QA after elution.

The desorption of a metal-loaded sorbent was investigated not only for the possibility of reusing of the sorbent but also for the recovery of the valuable metal ion. Firstly, different types of eluents were used for the desorption of W(VI) ions, such as ascorbic acid, HCl, NH_4_Cl, NaOH, and NH_4_OH solutions (0.25 M), which were used as eluting agents at room temperature for 10 min. The obtained data in Figure 16A show that the best eluent for the efficient recovery of W(VI) is NH_4_Cl, which is capable of recovering 79.2% of loaded W(VI) ions on HNAP/QA. Secondly, the influence of the concentration of NH_4_Cl on the recovery of W(VI) ions was then studied using several concentrations varying from 0.25 to 2.0 M, as shown in Figure 16B. The obtained data show that 0.5 M NH_4_Cl achieved a recovery of 92.6% of the loaded W(VI) ions on HNAP/QA. Lastly, the influence of the desorption time was assessed by ranging the elution period between 5 and 50 min. Figure 16C demonstrates that 20 min was sufficient for the recovery of almost 79.2% of the W(VI) ions from the loaded sorbent. It is completely clear that when the metal-loaded HNAP/QA was investigated before and after desorption with 0.5 M NH_4_Cl for 20 min at an ambient temperature, no presence of W(VI) ions was detected after desorption; it is an evident that all W(VI) ions were recovered back to the solution, as provided in Figure 17.

### 2.4. Recovery of W(VI) Ions from Wolframite Ore

One of the most important and promising areas is Gabal (G) Qash Amir, in the extreme southeastern part of Egypt. It is considered a rich area with economic and strategic metals such as Mn, Zr, Ta, U, Nb, and W. In the last decade, many new W-rich mineralizations were discovered in the studied area of new Gabal Qash Amir [40,41,42,43,44,45]. The region known as G. Qash Amir is in the extreme southeastern corner of the Egyptian Eastern Desert; it is located approximately 28 km southwest of Abu-Ramad city and not far from the border of Sudan, bounded by longitudes 36°10′59″–36°14′24″ E and latitudes 22°14′07″–22°15′21″ N. This area is a section of the Arabian-Nubian shield zone. 

#### 2.4.1. Pre-Concentration of Wolframite Ore

**Figure 18:**
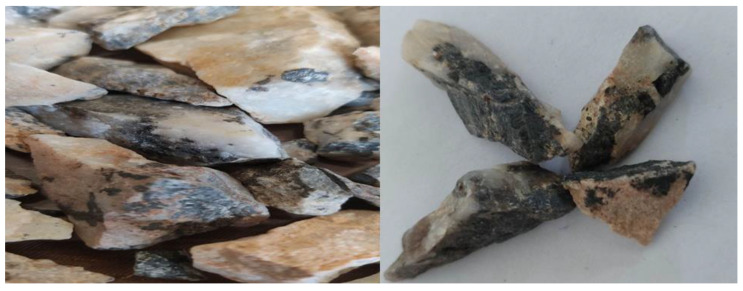
Photographs of selected wolframite sample and grains.

A working technological ore sample was precisely collected from a mineralized invading quartz vein within G. Qash Amir wolframite granite. A bulk sample (10 kg, containing around 0.43% *w*/*w* of WO_3_) was ground using a roll mill crusher and jaw crushers to diminish the mineral particles to a size of less than 1.0 mm. The gravity concentration was performed using a lab wet Wilfley shaking table (Holman-Wilfley Ltd., Redditch, UK); this operation allowed for the production of a W-rich concentrate (about 83.0 g). The next step in the pre-processing consisted of a magnetic separation utilizing a high-intensity induced magnetic roll separator (Carpco Model MLH (13) III-5, Outokumpu Technology, Inc., Jacksonville, FL, USA) to recover wolframite-rich minerals (Figure 18).

#### 2.4.2. Characterizations of the Wolframite Ore

**Figure 19:**
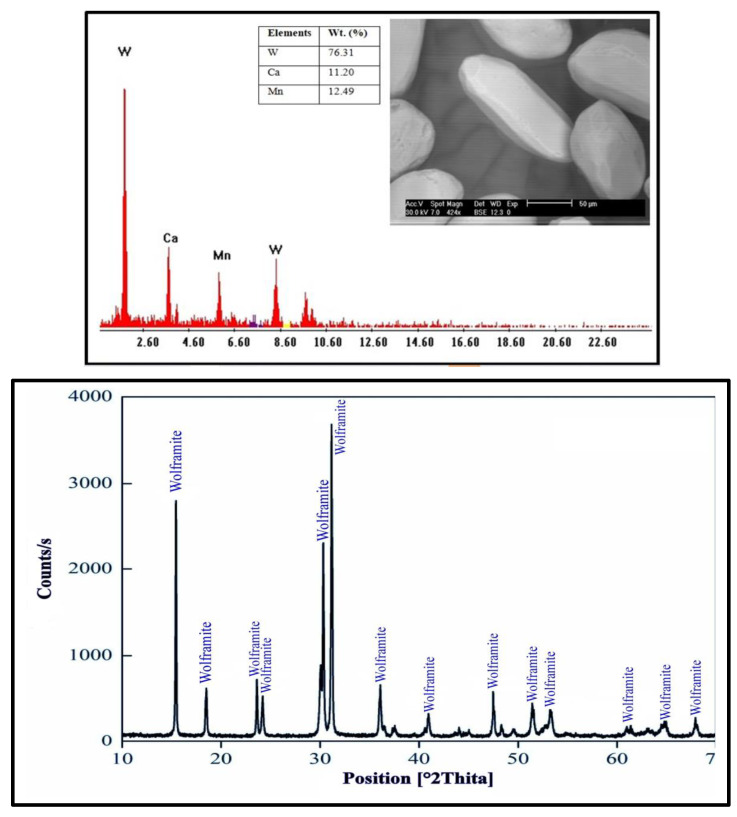
SEM-EDX and XRD analyses of wolframite sample.

Wolframite is a tungsten mineral that mainly consists of manganese tungsten oxide (Fe,Mn)WO_4_; it is the intermediate mineral between ferberite (Fe^2+^ rich) and hübnerite (Mn^2+^ rich). The semi-quantitative analysis of wolframite samples shows the preponderance of both W and Mn elements (88.8%, *w*/*w*) and the presence of a non-negligible fraction of silicate and iron. The wolframite sample was confirmed using SEM-EDX and XRD characterizations, as can be seen in Figure 19 and Table 5.

#### 2.4.3. Extraction of Tungsten Oxide

The first stage is the tungsten leaching from W-concentrate; this process was carried out with HCl (30% *w*/*w* HCl solution) to eliminate most of the Mn and Fe content [53]. The acid-leaching step was performed using continuous stirring at 400 rpm for 3 h at 110 °C and a 1:1.5 solid to liquid (S/L) ratio. Secondly, the wolframite sample was subjected to alkaline leaching using 300 g of NaOH (40%, *w*/*w*, NaOH solution); the leaching process was applied at 400 rpm and a S/L ratio of 1:3 for 2 h at 130 °C. The alkaline leaching process allowed for the dissolution of almost all the W-content and some traces of Mn and Fe. The obtained leachate was analyzed, and the W concentration is listed in Table 6.

**Figure 20:**
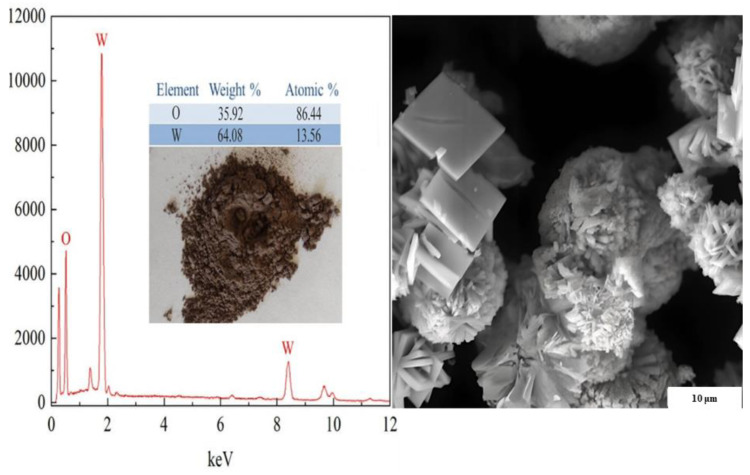
SEM-EDX analysis of obtained tungsten oxide product.

The leachate was filtered, and the pH was adjusted to 4.5. The HNAP/QA adsorbent was used to adsorb W(VI) ions from the leachate; the adsorption process was conducted based on the best controlling factors studied previously (pH 4.5, agitation time of 60 min, 2.0 g of HNAP/QA, and 250 mL of leachate at room temperature). Adsorption was accomplished, and the W(VI) content was recoverable through the separation of HNAP/QA in 0.5 M of NH_4_Cl for 20 min at room temperature. Later, the W(VI) ions were precipitated from the eluate using 30% HCl. A yellow WO_3_·nH_2_O product was formed. Finally, the precipitate was rinsed multiple times with ultrapure water and heated at 70–90 °C for 30 min to dry. This was followed by calcination at 500 °C for 2 h. The final product was characterized using SEM-EDX analysis, and the data are shown in Figure 20. 

**Figure 21:**
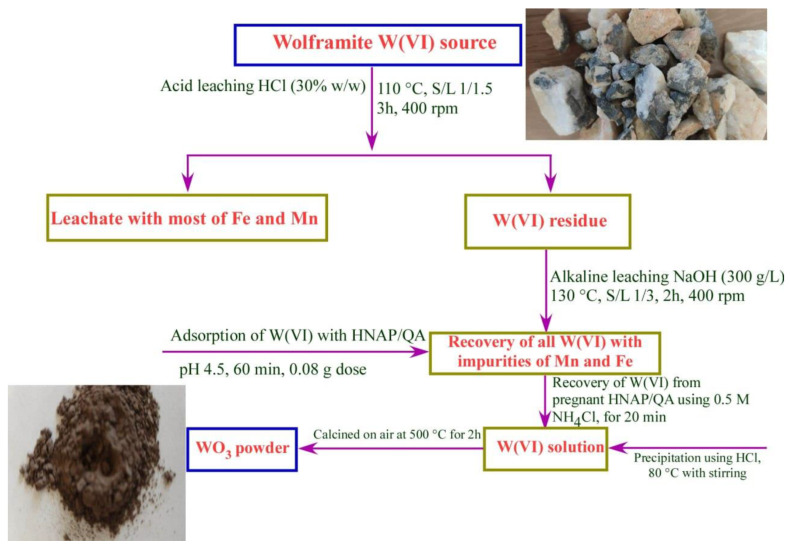
Flow sheet of the recovery of tungsten oxide from wolframite sample.

The EDX patterns indicate the W and O as the major elements and no notable impurities, while the SEM image shows the irregular shape of WO_3_ to be a flower-like structure. A flow sheet summarizing the recovery of W(VI) ions from the wolframite ore is provided in Figure 21.

## 3. Materials and Methods

### 3.1. Chemicals and Reagents

P-phenylenediamine and 2-hydroxy-5-nitrobenzenaldehyde were purchased from Thermo Fisher Scientific (Morris Plains, NJ, USA). Shanghai Makclin Biochemical Co., Ltd. (Shanghai, China) provided the glycidyl trimethyl ammonium chloride (95%), AlCl_3_, and NaOH. Phosphorus oxychloride, sodium tungstate (Na_2_WO_4_⋅2H_2_O), NaCl, CHCl_3_, CDCl_3_, dimethyl sulfoxide, and CH_3_OH were purchased from Sigma-Aldrich (St. Louis, MO, USA). All chemicals were of a very high purity level and required no further processing before use. All the experimental solutions were prepared using ultrapure water of 18.2 MΩ·cm. Thin paper chromatography (PC) was utilized to explore the synthesis process. Furthermore, a basic UV lamp set to 250 nm and an eluent comprising a combination of 1:1 *v*/*v* ethanol and ethyl acetate were used to identify spots formed on the PC.

### 3.2. Preparation of the Adsorbent (HNAP/QA)

**Scheme 1:**
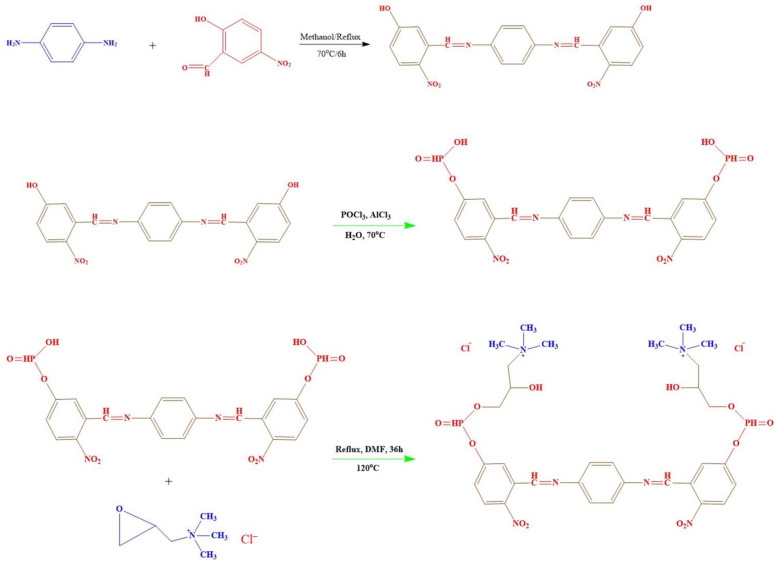
Preparation of HNAP and HNAP/QA.

A methanolic solution of 2-hydroxy-5-nitrobenzenaldehyde (10 mL, 25 mmol) was added drop-wise to a 10 mL methanolic solution of p-phenylenediamine (25 mmol) in the presence of an acid (2 mL of 98% H_2_SO_4_) with stirring. The mixture was allowed to reflux at 70 °C for 6 h. The reaction’s volume decreased by half under a vacuum. An orange-yellow precipitate was formed, which was filtered, washed out multiple times by methanol, and left to air-dry overnight. The TLC was used to monitor the progress of the condensation reaction. The produced Schiff base (3,3′-((1,4-phenylenebis(azaneylylidene))-bis(methaneylylidene))-bis(4-nitrophenol)) was provided a PZN abbreviation, and the final yield was determined as 90%. The produced material was subjected to phosphorylation, which took place through an interaction with 0.2 g of AlCl_3_ and 25 mL phosphorus oxychloride with vigorous stirring for 30 h at a temperature of 110 °C (Scheme 1). AlCl_3_ and POCl_3_ were used as phosphorylating agents to introduce a phosphonic acid group. The AlCl_3_ aids in the release of HCl during the reaction. A pale yellow precipitate was formed, separated through filtration, rinsed numerous times with ultrapure water and methanol, and allowed to dry overnight in the air. The produced material was named HNAP, referring to phosphorylated beads of (3-(((4-((5-(((S)-hydroxyhydrophosphoryl)oxy)-2-nitrobenzylidene) amino) phenyl) imino) methyl)-4-nitrophenyl hydrogen (R)-phosphonate)); its purity was assessed using TLC and was found to be 94.0%, (Scheme 1).

The phosphorylated beads (HNAP) were subjected to quaternization; this reaction occurred through the reflux of 4.5 g of HNAP in a three-necked flask containing a solution of 12.0 g of glycidyl trimethyl ammonium chloride with vigorous stirring for 36 h at 100 °C. The final product was separated by filtration and washed out several times with ultrapure water and methanol to remove the unreacted materials and solvent. The product was dried then overnight in the air. The produced material, HNAP/quaternary ammonium salt, was named (HNAP/QA). All synthesized chemicals were provided with names in accordance with the IUPAC nomenclature.

### 3.3. Instrumentation

We tested the adsorbent using the following spectroscopic techniques: FTIR (Nicolet™ iS50, Thermo Fischer Scientific, Morris Plains, NJ, USA), mass spectra using a Hitachi M-8000 unit, Tokyo, Japan, SEM-EDX using a Hitachi S 4160 FE SEM, Tokyo, Japan, and NMR in a 500 MHz field using a Bruker Fourier 80, Billerica, MA, USA. The concentrations of ions were measured using inductively coupled plasma mass spectrometry (ICP-MS) (NexION 1000, PerkinElmer, Waltham, MA, USA).

### 3.4. Adsorption and Elution Experiments

A tungstate stock solution of 1000 mg L^−1^ was prepared by dissolving a proper weight of sodium tungstate (Na_2_WO_4_⋅2H_2_O) in a 0.01 M NaCl solution. The sorption experiment was conducted in three independent batches, each of which was made from the stock solution. HCl and NaOH, at concentration of 0.1 M each, were administered to adjust the acidity of the solution. For the standard batch sorption tests, the experiments were carried out using a 20 mL of 250 mg L^−1^ W(VI) ion solution for 30 min and 0.1 g of HNAP/QA at 25 °C, unless stated otherwise. The pH of the aqueous medium was changed from 1.0 to 6.0 to study the impact of the pH value. In addition, the experiments were performed at an amount of adsorbent between 0.01 and 0.2 g for a period of 30 min and a 250 mg L^−1^ W(VI) ion solution at a pH of 4.5 and at room temperature to investigate the effect of the adsorbent dose. The sorption time was studied likewise by varying the contact time between 5 and 90 min. Finally, the effect of temperature was investigated between 25 and 70 °C. The adsorption efficiency (*S*%) of the W(VI) ions and the adsorption capacity (*q_e_*) were calculated using equations below:

(10) $$S\%=\frac{C_{o}-C_{e}}{C_{o}}\times 100$$

(11) $$q_{e}=(C_{o}-C_{e})\times \frac{V}{m}$$

where *C_o_* and *C_e_* (mg L^−1^) are the W(VI) ion concentrations in the liquid phase at the beginning and end of the experiment, respectively, and *q_e_* (mg g^−1^) symbolizes the sorption capacity. In this equation, *V* (L) represents the volume of the working solution, and *m* (g) corresponds to the dry weight of the HNAP/QA.

### 3.5. Desorption Experiments

Desorption is a crucial step in recycling a sorbent material and recovering W(VI) ions. Once the adsorption experiment was completed, desorption tests were performed using the adsorbent in a batch system. NaOH, NH_4_OH, HCl, NH_4_Cl, and ascorbic acid were among the desorptive substances investigated in this study. The desorption time and the concentration of the desorptive substances were also studied. The concentrations of the metal ions in the supernatant were detected using ICP-MS.

## 4. Conclusions

HNAP/QA, which is an abbreviation of (3-(((4-((5-(((S)-hydroxyhydrophosphoryl)oxy))-2-nitrobenzylidene) amino) phenyl) imino) methyl)-4-nitrophenyl hydrogen (R)-phosphonate), is a new synthetic adsorbent material. HNAP/QA has a high selectivity for W(VI) ions from its aqueous solution. Several characterization techniques, such as FTIR, GC-MS, TGA, and NMR analyses, were used to ensure its successful preparation. The optimal factors influencing the retention of W(VI) ions on the new adsorbent were thoroughly examined and found to be a pH of 4.5 and 30 min of sorption time at room temperature. Kinetics and thermodynamics were also investigated; the adsorption reaction follows a Langmuir model. The sorption process of the W(VI) ions is spontaneous due to the negative value of ∆*G*° for all temperature values, while the positive value of ∆*H*° indicates that the adsorption on HNAP/QA is an endothermic mechanism. In addition, the positive value of ∆*S*° proposes an increase in the randomness of the adsorption activities in the investigated system. Finally, HNAP/QA was successfully used for the adsorption of W(VI) ions from wolframite ore, and pure W(VI) ions were separated via precipitation.

## Figures and Tables

**Table 1:** Surface area and porosity of HNAP and HNAP/QA.

Substances	S_BET_ (m^2^ g^−1^)	Pore Volume (cm^3^ g^−1^)	Pore Size (nm)
HNAP	101.6	0.205	2.8
HNAP/QA	178.4	0.288	1.78

**Table 2:** Kinetic parameters of W(VI) ions adsorbed on HNAP/QA.

PFO Kinetic				PSO Kinetic		
*K*_1_	*q_e_*_(cal)_	R^2^	*q_e_*_(exp)_	*K*_2_	*q_e_*_(cal)_	R^2^
0.11469	110.535	0.9737	62.44	0.00145	66.225	0.9965

**Table 3:** Isotherms parameters of W(VI) ions adsorbed on HNAP/QA.

Models	Parameters	
Langmuir isotherm	Equation	y = 0.003x + 0.1832
	*q_max_* (mg g^−1^)	333.33
	*K*_1_	0.0164
	R²	0.9937
Freundlich isotherm	Equation	y = 0.3982x + 1.5077
	*K_f_* (mg g^−1^)	32.189
	1/*n* (mg min g^−1^)	0.3982
	R²	0.9894
D-R isotherm	Equation	y = −0.0064x + 5.7831
	*q_D_* (mg g^−1^)	324.76
	*B_D_* (Mol^2^ kJ^−2^)	0.0064
	*E* (kJ mol^−1^)	8.8388
	R²	0.9621
Practical Capacity	*q_e_*_(exp)_ (mg g^−1^)	326.75

**Table 4:** Thermodynamic parameters of W(VI) ions adsorbed on HNAP/QA.

Δ*S*° (J mol^−1^ K^−1^)	Δ*H*° (kJ mol^−1^)	Δ*G*° (kJ mol^−1^)				
75.887	10.36	298 K	313 K	323 K	333 K	343 K
		−21.89	−22.81	−23.54	−24.27	−25.0

**Table 5:** Chemical compositions of wolframite sample.

Element	Concentration (%)
WO_3_	77.13
MnO	8.99
SiO_2_	0.58
Al_2_O_3_	0.3
Fe_2_O_3_	3.9
SO_3_	0.49
CaO	7.57
Others	1.04

**Table 6:** Chemical compositions of wolframite leachate.

Element	Concentration (%)
WO_3_	89.46
MnO	0.004
SiO_2_	0.46
Al_2_O_3_	0.3
Fe_2_O_3_	0.002
CaO	7.57

## Data Availability

Not applicable.

## Supplementary Materials

The following supporting information can be downloaded at: www.mdpi.com/article/10.3390/ijms24087423/s1. References [54,55] are cited in the supplementary material.

## References

[1] Liu H., Liu H., Nie C., Zhang J., Steenari B., Ekberg C. (2020). Comprehensive treatments of tungsten slags in China: A critical review. J. Environ. Manag..

[2] Shen L., Li X., Lindberg D., Taskinen P. (2019). Tungsten extractive metallurgy: A review of processes and their challenges for sustainability. Miner. Eng..

[3] Datta S., Vero S.E., Hettiarachchi G.M., Johannesson K. (2017). Tungsten contamination of soils and sediments: Current state of science. Curr. Pollut. Rep..

[4] Koutsospyros A., Strigul N., Braida W., Christodoulatos C. (2011). Tungsten: Environmental Pollution and Health Effects. Encyclopedia of Environmental Health.

[5] Ünal Ü., Somer G. (2012). A new and very simple procedure for the differential pulse polarographic determination of ultra trace quantities of tungsten using catalytic hydrogen wave and application to tobacco sample. J. Electroanal. Chem..

[6] Seiler R.L., Stollenwerk K.G., Garbarino J.R. (2005). Factors controlling tungsten concentrations in ground water Carson Desert, Nevada. Appl. Geochem..

[7] Bednar A., Mirecki J., Inouye L., Winfield L., Larson S., Ringelberg D. (2007). The determination of tungsten, molybdenum, and phosphorus oxyanions by high performance liquid chromatography inductively coupled plasma mass spectrometery. Talanta.

[8] Li S., Deng N., Zheng F., Huang Y. (2003). Spectrophotometric determination of tungsten (VI) enriched by nanometer-size titanium dioxide in water and sediment. Talanta.

[9] Bazel Y., Lešková M., Rečlo M., Šandrejová J., Simon A., Fizer M., Sidey V. (2018). Structural and spectrophotometric characterization of 2-[4-(dimethylamino) styryl]-1-ethylquinolinium iodide as a reagent for sequential injection determination of tungsten. Spectrochim. Acta Part A Mol. Biomol. Spectrosc..

[10] Chen W., Yang W.Z., Liu X.Q., Chen X.M. (2016). Structural, dielectric and magnetic properties of Ba3SrLn2Fe2Nb8O30(Ln = La, Nd, Sm) filled tungsten bronze ceramics. J. Alloys Compd..

[11] Chong X., Jiang Y., Zhou R., Feng J. (2016). Stability, chemical bonding behavior, elastic properties and lattice thermal conductivity of molybdenum and tungsten borides under hydrostatic pressure. Ceram. Int..

[12] Ravi U.K., Kumar J., Kumar V., Sankaranarayana M., Nageswara Rao G.V.S., Nandy T.K. (2016). Effect of cyclic heat treatment and swaging on mechanical properties of the tungsten heavy alloys. Mater. Sci. Eng. A.

[13] Stanciu V.I., Vitry V., Delaunois F. (2016). Tungsten carbide powder obtained by direct carburization of tungsten trioxide using mechanical alloying method. J. Alloys Compd..

[14] Medici S., Costa M., Brocato J., Peana M., Oksuz B.A., Cartularo L., Vaughan J., Laulicht F., Wu F., Kluz T. (2015). Tungsten-induced carcinogenesis in human bronchial epithelial cells. Toxicol. Appl. Pharmacol..

[15] Chen Y., Huo G., Guo X., Wang Q. (2022). Sustainable extraction of tungsten from the acid digestion product of tungsten concentrate by leaching-solvent extraction together with raffinate recycling. J. Clean. Prod..

[16] Huo G., Peng C., Song Q., Lu X. (2014). Tungsten removal from molybdate solutions using ion exchange. Hydrometallurgy.

[17] Fu F., Wang Q. (2011). Removal of heavy metal ions from wastewaters: A review. J. Environ. Manag..

[18] Kim J.W., Lee W.G., Hwang I.S., Lee J.Y., Han C. (2015). Recovery of tungsten from spent selective catalytic reduction catalysts by pressure leaching. J. Ind. Eng. Chem..

[19] Qin W., Xi X., Zhang L., Nie Z., Liu C., Li R. (2023). The effect of MOx (M = Zr, Ce, La, Y) additives on the electrochemical preparation of tungsten in eutectic Na_2_WO_4_–WO_3_ melt. J. Electroanal. Chem..

[20] Han Z., Golev A., Edraki M. (2021). A Review of Tungsten Resources and Potential Extraction from Mine Waste. Minerals.

[21] Wu X., Zhang G., Zeng L., Zhou Q., Li Z., Zhang D., Cao Z., Guan W., Li Q., Xiao L. (2019). Study on removal of molybdenum from ammonium tungstate solutions using solvent extraction with quaternary ammonium salt extractant. Hydrometallurgy.

[22] Zhao Z., Cao C., Chen X. (2011). Separation of macro amounts of tungsten and molybdenum by precipitation with ferrous salt. Trans. Nonferrous Met. Soc..

[23] Cao F., Wang W., Wei D., Liu W. (2021). Separation of tungsten and molybdenum with solvent extraction using functionalized ionic liquid tricaprylmethylammonium bis(2,4,4-trimethylpentyl) phosphinate. Int. J. Miner. Metall. Mater..

[24] Xu C., Zhan W., Tang X., Mo F., Fu L., Lin B. (2018). Self-healing chitosan/vanillin hydrogels based on Schiff-base bond/hydrogen bond hybrid linkages. Polym. Test..

[25] Pandya H.J., Ganatra K.J. (2008). Synthesis, characterization and biological evaluation of bis-bidentate Schiff base metal complexes. Inorg. Chem. Indian J..

[26] Li L., Sakr A.K., Schlöder T., Klein S., Beckers H., Kitsaras M.-P., Snelling H.V., Young N.A., Andrae D., Riedel S. (2021). Searching for Monomeric Nickel Tetrafluoride: Unravelling Infrared Matrix Isolation Spectra of Higher Nickel Fluorides. Angew. Chem. Int. Ed..

[27] Cai Y., Wu C., Liu Z., Zhang L., Chen L., Wang J., Wang X., Yang S., Wang S. (2017). Fabrication of a phosphorylated graphene oxide–chitosan composite for highly effective and selective capture of U(VI). Environ. Sci. Nano.

[28] Belfer S., Fainchtain R., Purinson Y., Kedem O. (2000). Surface characterization by FTIR-ATR spectroscopy of polyethersulfone membranes-unmodified, modified and protein fouled. J. Membr. Sci..

[29] Lin-Vien D., Colthup N.B., Fateley W.G., Grasselli J.G., Lin-Vien D., Colthup N.B., Fateley W.G., Grasselli J.G. (1991). CHAPTER 10—Compounds Containing –NH_2_, –NHR, and –NR_2_ Groups. The Handbook of Infrared and Raman Characteristic Frequencies of Organic Molecules.

[30] Hamza M.F., Salih K.A.M., Zhou K., Wei Y., Abu Khoziem H.A., Alotaibi S.H., Guibal E. (2022). Effect of bi-functionalization of algal/polyethyleneimine composite beads on the enhancement of tungstate sorption: Application to metal recovery from ore leachate. Sep. Purif. Technol..

[31] Sethuraman P.R., Leparulo M.A., Pope M.T., Zonnevijlle F., Brevard C., Lemerle J. (1981). Heteropolypentatungstobisphosphonates —Cyclopentane-like pseudorotation of an oxometalate structure. J. Am. Chem. Soc..

[32] Bajaber M.A., Ragab A.H., Sakr A.K., Atia B.A., Fathy W.M., Gado M.A. (2023). Application of a new derivatives of traizole Schiff base on chromium recovery from its wastewater. Sep. Sci. Technol..

[33] Lagergren S. (1898). About the theory of so-called adsorption of soluble substances, Zur theorie der sogenannten adsorption geloster stoffe. K. Sven. Vetensk. Handl..

[34] Sakr A.K., Abdel Aal M.M., Abd El-Rahem K.A., Allam E.M., Abdel Dayem S.M., Elshehy E.A., Hanfi M.Y., Alqahtani M.S., Cheira M.F. (2022). Characteristic Aspects of Uranium (VI) Adsorption Utilizing Nano-Silica/Chitosan from Wastewater Solution. Nanomaterials.

[35] Negm S.H., Abd El-Hamid A.A.M., Gado M.A., El-Gendy H.S. (2018). Selective uranium adsorption using modifed acrylamide resins. J. Radioanal. Nucl. Chem..

[36] Washahy A.R., Sakr A.K., Gouda A.A., Atia B.M., Somaily H.H., Hanfi M.Y., Sayyed M.I., El Sheikh R., El-Sheikh E.M., Radwan H.A. (2022). Selective Recovery of Cadmium, Cobalt, and Nickel from Spent Ni-Cd Batteries using Adogen® 464 and Mesoporous Silica Derivatives. Int. J. Mol. Sci..

[37] Wang J., Guo X. (2020). Adsorption kinetic models: Physical meanings, applications, and solving methods. J. Hazard. Mater..

[38] Ibrahium H.A., Atia B.M., Awwad N.S., Nayl A.A., Radwan H.A., Gado M.A. (2022). Efficient preparation of phosphazene chitosan derivatives and its applications for the adsorption of molybdenum from spent hydrodesulfurization catalyst. J. Dispers. Sci. Technol..

[39] Ruiping L., Chunye L., Xitao L. (2006). Adsorption of tungstate on kaolinite: Adsorption models and kinetics. RSC Adv..

[40] Alharbi A., Gouda A.A., Atia B.M., Gado M.A., Alluhaybi A.A., Alkabli J. (2022). The Role of Modified Chelating Graphene Oxide for Vanadium Separation from Its Bearing Samples. Russ. J. Inorg. Chem..

[41] Atia B.M., Sakr A.K., Gado M.A., El-Gendy H.S., Abdelazeem N.M., El-Sheikh E.M., Hanfi M.Y., Sayyed M.I., Al-Otaibi J.S., Cheira M.F. (2022). Synthesis of a New Chelating Iminophosphorane Derivative (Phosphazene) for U (VI) Recovery. Polymers.

[42] Atia B.M., Khawassek Y.M., Hussein G.M., Gado M.A., El-Sheify M.A., Cheira M.F. (2021). One-pot synthesis of pyridine dicarboxamide derivative and its application for uranium separation from acidic medium. J. Environ. Chem. Eng..

[43] Atia B.M., Gado M.A., Cheira M.F., El-Gendy H.S., Yousef M.A., Hashem M.D. (2021). Direct synthesis of a chelating carboxamide derivative and its application for thorium extraction from Abu Rusheid ore sample, South Eastern Desert, Egypt. Int. J. Environ. Anal. Chem..

[44] Gado M., Rashad M., Kassab W., Badran M. (2021). Highly Developed Surface Area Thiosemicarbazide Biochar Derived from Aloe Vera for Efficient Adsorption of Uranium. Radiochemistry.

[45] Akar T., Kaynak Z., Ulusoy S., Yuvaci D., Ozsari G., Akar S.T. (2009). Enhanced Biosorption of Nickel (II) Ions by Silica-GelImmobilized Waste Biomass: Biosorption Characteristics in Batch and Dynamic Flow Mode. J. Hazard Mater..

[46] Chen A.H., Chen S.M. (2009). Biosorption of Azo Dyes from Aqueous Solution by Glutaraldehyde-Crosslinked Chitosans. J. Hazard Mater..

[47] Dubinin M.M. (1960). The Potential Theory of Adsorption of Gases and Vapors for Adsorbents with Energetically Nonuniform Surfaces. Chem. Rev..

[48] Mittal A., Kaur D., Mittal J. (2009). Batch and Bulk Removal of a Triarylmethane Dye, Fast Green FCF, from Wastewater by Adsorption over Waste Materials. J. Hazard Mater..

[49] Chen A.H., Yang C.Y., Chen C.Y., Chen C.Y., Chen C.W. (2009). The Chemically Crosslinked Metal-Complexed Chitosans for Comparative Adsorptions of Cu (II), Zn (II), Ni (II) and Pb (II) Ions in Aqueous Medium. J. Hazard Mater..

[50] Kundu S., Gupta A.K. (2006). Arsenic Adsorption onto Iron OxideCoated Cement (IOCC): Regression Analysis of Equilibrium Data with Several Isotherm Models and Their Optimization. Chem. Eng. J..

[51] Hassanin M.A., Negm S.H., Youssef M.A., Sakr A.K., Mira H.I., Mohammaden T.F., Al-Otaibi J.S., Hanfi M.Y., Sayyed M.I., Cheira M.F. (2022). Sustainable Remedy Waste to Generate SiO_2_ Functionalized on Graphene Oxide for Removal of U(VI) Ions. Sustainability.

[52] Gado M., Atia B. (2019). Adsorption of Thorium Ions Using Trioctylphosphine Oxide Impregnated Dowex 1 × 4 Powder. Radiochemistry.

[53] Weshahy A.R., Gouda A.A., Atia B.M., Sakr A.K., Al-Otaibi J.S., Almuqrin A., Hanfi M.Y., Sayyed M.I., El Sheikh R., Radwan H.A. (2022). Efficient Recovery of Rare Earth Elements and Zinc from Spent Ni–Metal Hydride Batteries: Statistical Studies. Nanomaterials.

[54] Borodina E., Karpov S.I., Selemenev V.F., Schwieger W., Maracke S., Fröba M., Rößner F. (2015). Surface and texture properties of mesoporous silica materials modified by silicon-organic compounds containing quaternary amino groups for their application in base-catalyzed reactions. Microporous Mesoporous Mater..

[55] Tarasova N.P., Smetannikov Y.V., Zanin A.A. (2013). Radiation-chemical transformation of elemental phosphorus in the presence of ionic liquids. Dokl. Chem..

