# Supplementary material for: Recovery of W(VI) from Wolframite Ore Using New Synthetic Schiff Base Derivative
Source: Int J Mol Sci. 2023 Apr 18;24(8):7423. doi: 10.3390/ijms24087423 (PMC10139163; doi:10.3390/ijms24087423)
Supplement: Supplementary file 1 [file ijms-24-07423-s001.zip › ijms-2287060-supplementary.pdf]

Recovery of W(VI) From Wolframite Ore Using New Synthetic Schiff Base Derivative

Rawan E. Elbshary[1], Ayman A. Gouda[2], Ragaa El Sheikh[2], Mohammed S. Alqahtani[3,4,5], Mohamed Y. Hanfi[6,7], Bahig M. Atia[6], Ahmed K. Sakr[8,*] and Mohamed A. Gado[6,*]

[1] Department of Chemistry, Faculty of pharmacy, Heliopolis University, El Salam City, Cairo 11785, Egypt
[2] Department of Chemistry, Faculty of Science, Zagazig University, Zagazig 44519, Egypt
[3] Radiological Sciences Department, College of Applied Medical Sciences, King Khalid University, Abha 61421, Saudi Arabia
[4] BioImaging Unit, Space Research Centre, University of Leicester, Michael Atiyah Building, LE1 7RH, Leicester, UK
[5] Research Center for Advanced Materials Sciences (RCAMS), King Khalid University, Abha 61413, Saudi Arabia
[6] Nuclear Materials Authority, El Maadi, Cairo P.O. Box 530, Egypt
[7] Institute of Physics and Technology, Ural Federal University, St. Mira, 19, 620002 Yekaterinburg, Russia
[8] Department of Civil and Environmental Engineering, Wayne State University, 5050 Anthony Wayne Drive, Detroit, MI 48202, USA

* Correspondence: akhchemist@gmail.com (A.K.S.); mag.nma@yahoo.com (M.A.G.)

1. Thermal Analysis

The thermal degradation of amine groups and carboxylic groups in Figure S1a demonstrates that the first peak at maxima 100–228 °C corresponds to the removal of physically adsorbed and internal water, the second peak at 228–452 °C was assigned to the elimination of quaternary amine groups [1]. The peak at 670–890 °C along with the appearance of a new final weight residue stage may be due to the formation of the residual phosphorous oxide [2]. The stability of the HNAP/QA increased due to phosphorylation; the grafting of phosphorous element is a well-known method for increasing the thermal stability of fire-retardant materials.

As seen in the DSC analysis (Fig. S1b), the substituents bearing the quaternary amine groups are probably degraded in the temperature range 450–500 °C. the quaternary amine groups degradation peak appears at 483 °C [45, 55].

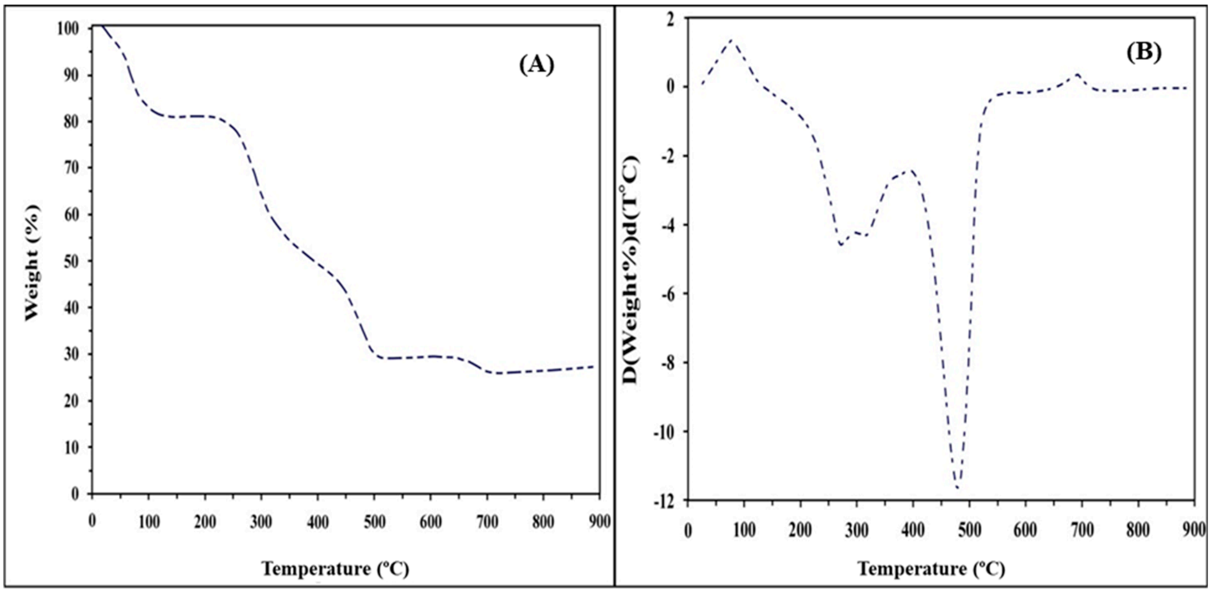

Fig. (S1): (A) TGA analysis of HNAP/QA and (B) DSC analysis of HNAP/QA.

References:

[45] Borodina, E.; Karpov, S.I.; Selemenev, V.F.; Schwieger, W.; Maracke, S.; Fröba, M.; Rößner, F. Surface and texture properties of mesoporous silica materials modified by silicon-organic compounds containing quaternary amino groups for their application in base-catalyzed reactions. *Microporous and Mesoporous Mater*. **2015**, *203*, 224-231. doi: 10.1016/j.micromeso.2014.10.009.

[55] Tarasova, N.P., Smetannikov, Y.V.; Zanin, A.A. Radiation-chemical transformation of elemental phosphorus in the presence of ionic liquids. *Dokl. Chem***. 2013**, 449, 111–113. doi: 10.1134/S0012500813040010
